# Atypical focal xanthogranulomatous pyelonephritis without clinical symptoms presenting as infiltrative renal cancer: a case report and literature review

**DOI:** 10.1186/s12894-020-00632-3

**Published:** 2020-06-03

**Authors:** Xiaobo Ding, Gang Wang, Tiejun Wang, Xiaobo Ma, Yanbo Wang

**Affiliations:** 1 Department of Radiology, First Hospital of Jilin University, Changchun, Jilin 130021 P.R. China; 2 Second Operating Room, First Hospital of Jilin University, Changchun, Jilin 130021 P.R. China; 3 Department of Orthopedic Traumatology, First Hospital of Jilin University, Changchun, Jilin 130021 P.R. China; 4 Department of Pathology, First Hospital of Jilin University, Changchun, Jilin 130021 P.R. China; 5 Department of Urology, First Hospital of Jilin University, Changchun, Jilin 130021 P.R. China

**Keywords:** Xanthogranulomatous pyelonephritis, Infiltrative renal cancer, Renal cancer, Kidney, Case report

## Abstract

**Background:**

Xanthogranulomatous pyelonephritis (XGP) is an uncommon form of chronic pyelonephritis. Most patients of XGP are diffuse in radiology and the clinical features are typical.

**Case presentation:**

We present a case of 24-year-old female with the absence of symptoms and normal laboratory examinations. Contrast computed tomography and intravenous pyelography demonstrate infiltrative renal mass and renal cell carcinoma is presumed. Laparoscopic right radical nephrectomy is performed, but the final pathological result shows XGP.

**Conclusions:**

As far as we know, this is the first case report of XGP without any symptoms/signs and with normal laboratory examinations. The diagnosis of atypical XGP is challenging and preoperative renal mass biopsy should be considered in special cases.

## Background

Xanthogranulomatous pyelonephritis (XGP) is one kind of chronic pyelonephritis and assumed as a complication of urinary tract obstruction or bacterial infection [1]. Typical characteristics of XGP are destruction of normal renal parenchyma and replacement of xanthoma cells [1, 2]. XGP has been reported from neonate to 84 years old patient and middle aged women are more common [1, 3]. Presence of clinical symptoms and typical radiologic features exist in most of XGP patients [4, 5]. Typical clinical symptoms of XGP patients are fever of unknown origin, abdomen/flank pain, weight loss, anemia or palpable renal mass, which are presented in most of the reported cases. Korkes F and colleagues retrospectively reviewed 41 cases of XGP, all patients were symptomatic [6]. Laboratory tests changes, C-reactive protein or leukocytosis, are shown in 88% of all patients [7]. The present report describes a rare case who presented without any symptoms and with normal laboratory findings, whose radiologic features show renal cancer.

## Case presentation

**Fig. 1:**
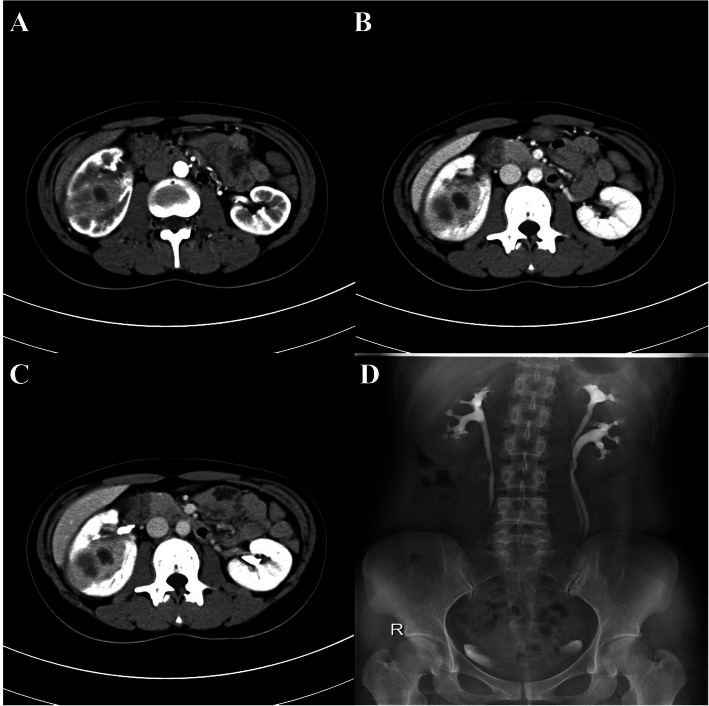
Contrast-enhanced CT scan showing a 6.0 cm × 4.9 cm heterogeneous enhancing mass with hypodense areas within it in the middle of right kidney (**a**, **b** and **c**). Intravenous pyelogram showing absence of dilatation or filling defects in the right renal calyces or pelvis (**d**)

**Fig. 2:**
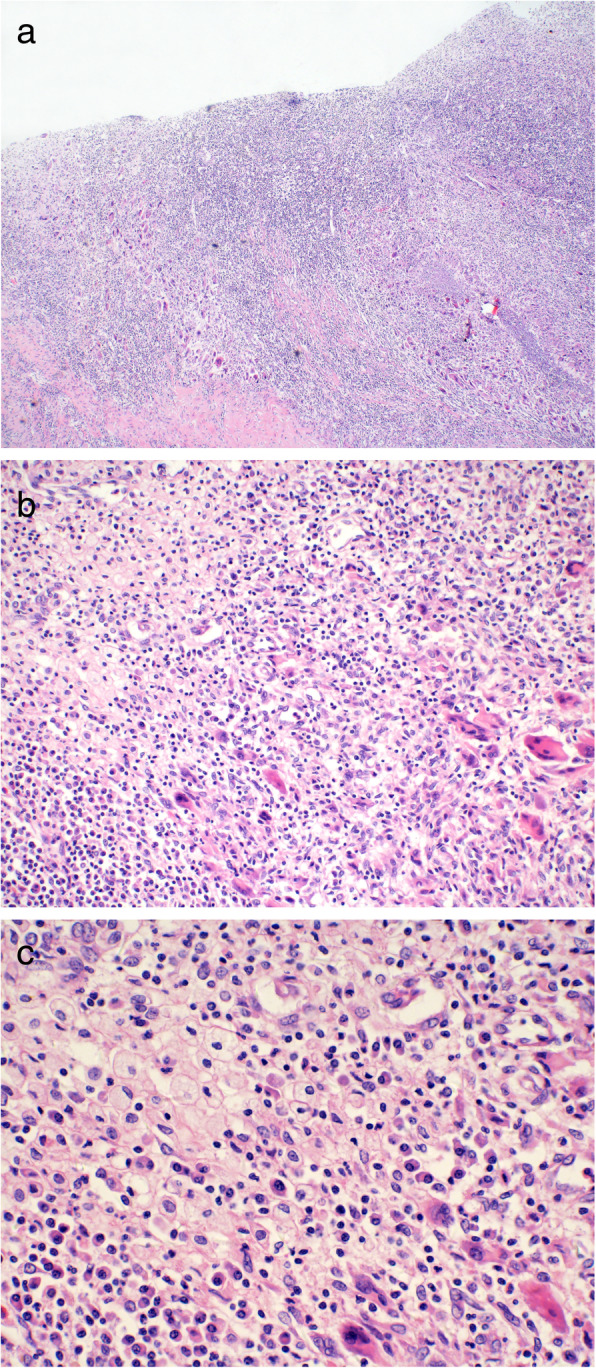
Microscopically, the majority of urothelium of the renal pelvis and calyceal is necrotic and scaled (**a**). At low power, the kidney shows a granulomatous inflammation with a large number of chronic inflammatory cell infiltration and focal abscess formation (hematoxylin-eosin, original magnification × 4) (**b**). At high power, the inflammatory infiltration is composed of a large number of lipid-laden xanthomatous cells, multinucleated giant cells, lymphoplasmacytic as well as some neutrophile cells (hematoxylin-eosin, original magnification × 40) (**c**)

A 24-year-old unmarried woman was incidentally admitted to our hospital during routine ultrasound examination, which showing a homogeneous solid-cystic mass with internal echoes and without well-defined margins in the right kidney. The patient was healthy and refused history of hematuria, frequency, fever, abdominal/flank pain and weight loss. Physical examination and routine laboratory tests were both normal. Contrast-enhanced computed tomography (CT) scan of the abdomen revealed a heterogeneous enhancing right renal mass with internal hypodense lesion with a diameter of 6.0 cm × 4.9 cm (Fig. 1a, b and c). Renal cancer was clinical considered but urothelial carcinoma was also possible. Intravenous pyelogram (IVP) demonstrated absence of dilatation or filling defects in the calyces (Fig. 1d). Infiltrative renal cell carcinoma was presumed and retroperitoneal laparoscopic right radical nephrectomy was performed. Pathologic examination reported macroscopically 5.5 cm × 5 cm × 3 cm sized yellow-white lesion and microscopically a granulomatous inflammation with a large number of lipid-laden xanthomatous cells, multinucleated giant cells lymphoplasmacytic as well as some neutrophile cells, supporting a diagnosis of XGP (Fig. 2). Immunohistochemical examination was not necessary based on the typical pathological characteristics of hematoxylin-eosin staining. The patient recovered uneventfully after surgery. Ultrasound was performed every year and the patient went well at follow-up 3 year later.

## Discussion and conclusions

XGP is an uncommon chronic inflammation of renal parenchyma, firstly described in 1916 [8]. XGP occurs approximately 1% of pyelonephritis in adults and 16% of pediatric nephrectomy specimens [9, 10]. Recently, Stoica I and colleagues retrospectively reviewed XGP cases from 1963 to 2016. Sixty-three patients (95.5%) underwent nephrectomy and 3 patients (4.5%) underwent partial nephrectomy [4]. XGP is frequently unilateral and bilateral cases of XGP are extremely rare. Shah K and colleagues reported one bilateral XGP child case managed non-surgically [11]. Hyla-Klekot L and colleagues reported one bilateral XGP patient managed by intensive therapy and partial nephrectomy [12].

The exact etiology of XGP is unclear and most of common associated factors are long term urinary tract obstruction or infection. Renal calculi, frequently staghorn stones, may be seen in up to 100% of the published cases [6]. Altered immune response and intrinsic disturbance of leukocyte function are other possible factors [1, 5, 10].

CT scan is priority for preoperative evaluation of XGP. Based on CT findings, XGP can be divided into diffuse type (92%) or focal type (8%) [13, 14]. Typical CT features of diffuse XGP patients are destruction of renal parenchyma and replacement by multiple, low-attenuation lesions with strong enhancement, described as “bear paw sign”. Depending on the extension of inflammation, XGP can be classified as three stages: nephric XGP (stage I), perinephric XGP (stage II) and paranephric XGP (stage III). In focal XGP patients, CT frequently reveal a well-defined localized intra renal lesion with hypo-attenuation [15].

The management of XGP is different for diffuse versus focal. Nephrectomy is the standard treatment approach for diffuse XGP patients, while medical therapy with antibiotics or nephron sparring surgery is priority for focal XGP patients. In the study of Korkes F et al., all 41 cases of XGP underwent nephrectomy [6]. In the study of Çaliskan S et al., one patient underwent partial nephrectomy and 12 patients were performed nephrectomy in all 13 cases of XGP [5].

In the study of Korkes F et al, 41 cases of XGP were retrospectively reviewed and all patients were symptomatic [6]. In the study of Chlif et al., 12 pseudotumoural XGP cases were reviewed [13]. An obstructive renal stone was shown in nine patients and one patient presented with loin pain. Blood investigations showed higher C-reactive protein in one patient and gram-negative organisms in four patients. In our study, the present case did not have any abnormal clinical symptoms and/or signs. Routine laboratory tests were normal. CT and IVP revealed renal cancer rather than XGP. Atypical clinical and radiologic characteristics make the preoperatively correct diagnosis difficult. In order to avoid misdiagnosis and mistreatment, preoperative renal mass biopsy is priority, although the inevitable false negative results were objectively existed [16]. Fitouri Z reported a XGP case which was confirmed by percutaneous renal lesion biopsy. The patient was successfully treated with 8 weeks’ antibiotic therapy [17]. Ho CI et al. also reported a XGP patient diagnosed by renal mass biopsy, who recovered by antibiotic therapy for 2 months [18]. Renal tumor biopsy can be used for treatment decision making, especially when a mass is suspected to be infectious, hematologic or metastatic [19, 20]. However, the indications of preoperative needle biopsy are still unclear and vary among centers.

In summary, we reported a case of focal XGP without typical clinical and radiological characteristics, which can mimic infiltrative renal cancer. In special cases, preoperative renal mass biopsy could be performed to avoid misdiagnosis and mistreatment.

## Data Availability

The data used in the current study are available from the corresponding author on reasonable request.

## Abbreviations

CT: Computed tomography
IVP: Intravenous pyelogram
XGP: Xanthogranulomatous pyelonephritis
